# Study on the Structure and Bioactivity of *Ganoderma lucidum* Polysaccharides under Cassava Stalk Stress

**DOI:** 10.3390/jof9050514

**Published:** 2023-04-26

**Authors:** Yijun Liu, Biyi Mai, Zhiyun Li, Xingqin Feng, Yunlan Chen, Lijing Lin, Qiuyu Xia

**Affiliations:** 1 Hainan Key Laboratory of Storage & Processing of Fruits and Vegetables, Agricultural Products Processing Research Institute, Chinese Academy of Tropical Agricultural Sciences, No. 48 Renmindadaonan, Zhanjiang 524001, China; 2 College of Food Science and Technology, Guangdong Ocean University, No. 1 Haida Road, Mazhang District, Zhanjiang 524088, China; 3 College of Tropical Crops Institute, Yunnan Agricultural University, Pu’er 650201, China; 4 Key Laboratory of Tropical Crop Products Processing of the Ministry of Agriculture and Rural Affairs, Zhanjiang 524001, China

**Keywords:** cassava stalk, *Ganoderma lucidum*, polysaccharide, structure, antioxidant activity in vitro

## Abstract

Various carbon sources affect the growth of the *G. lucidum* fruiting body, and the cassava stalk is considered a promising carbon source for *G. lucidum*. The composition, functional group characteristics, molecular weight distribution, antioxidant activity in vitro, and growth effect of *L. rhamnosus* LGG of *G. lucidum* polysaccharides (GLPs) under cassava stalk stress were investigated by gas chromatography-mass spectrometry, near-infrared spectroscopy, and gel chromatography. The results showed that GLPs consisted of D-glucose, D-galactose, and seven other monosaccharides. The end of the sugar chain had β-D-Glc and β-D-Gal configurations. The total sugar content in GLP1 was the highest (4.07%), and GLP1, GLP2, GLP3, and GLP5 had the β-D-Gal configuration, while GLP4 and GLP6 had the β-D-Glc configuration. The greater the proportion of cassava stalk, the greater the maximum molecular weight of GLPs. The total antioxidant capacities of GLPs obtained from different cassava stalks significantly varied, as well as their stimulating effects on the *L. rhamnosus* LGG growth. Higher concentrations of GLPs corresponded to the more intensive growth of *L. rhamnosus* LGG. This study provided essential data support for cassava stalk as a carbon source in *G. lucidum* cultivation.

## 1. Introduction

Cassava (*Manihot esculenta* Crantz) is an essential crop in tropical areas and plays a significant role in economic growth in tropical areas. However, the value-added utilization of cassava stalks after harvesting has always been a complex problem in the green development of the industry. Cassava stalks are as high as 7.5–10.5 t/ha, and the utilization rate is less than 10%. Most of them are burned on the spot or manually moved to the fields and abandoned by the roadside, resulting in an enormous waste of resources and environmental pollution. Cassava stems contain 40–50% cellulose and 25–30% starch [1,2], which are very beneficial to the growth of edible fungi. Cassava stalks have been successfully used to cultivate *Auricularia auricula* [3] and *Pleurotus geesteranus* [4] and have achieved good results.

*G. lucidum* is an essential medicinal fungus [5] and the main cultivated variety in China. The *G. lucidum* fruiting body contains polysaccharides, triterpenoids, alkaloids, and other active components, among which *G. lucidum* polysaccharides (GLPs) have been proven to regulate immunity, exert antitumor effects, reduce blood lipids, and resist chemical and immune liver injury [6,7]. GLPs are mainly composed of glucose, galactose, arabinose, mannose, xylose, fucose, and rhamnose, among which glucose, galactose, and mannose are the main monosaccharides, and the monosaccharide compositions of GLPs vary with different carbon sources [8,9]. The linkage of monosaccharides in GLPs is relatively complicated, with 1 → 2, 1 → 3, 1 → 4, and 1 → 6 linkages, and most of them are linked by 1→3 and 1→6 linkages. These glycosidic bonds have two conformations of α or β, among which β-polysaccharides play a significant role in biological activity [10,11]. The molecular weight of GLPs ranges from several thousand to several million Da, and the effects of GLPs with different molecular weight distribution ranges on antioxidant activity and biological activity in vitro vary [12,13]. *L. rhamnosus*, as normal flora in the human body, balances and improves gastrointestinal function, enhances human autoimmune ability, and so on [14]. Polysaccharides help promote the growth of *L. rhamnosus* [15]. However, there are quite a few reports on GLPs promoting the growth of *L. rhamnosus*, which limits the more comprehensive application of *G. lucidum* polysaccharides.

The biological activities of GLPs in immunomodulation, antitumor activity, blood lipid regulation, and liver protection are closely related to their structure and molecular characteristics [16,17]. The β-glucan GSP-2 isolated from GLPs can stimulate the proliferation of B lymphocytes in the mouse spleen, activate the phagocytic function of RAW264.7 macrophages and promote NO release [18]. GLPs can help treat cancer and improve immunity by regulating the secretion of IL-2, IL-6, and IFN-γ cytokines [19], changing the characteristics of intestinal flora and regulating the gene expression of colon epithelial cells [20], regulating the secretion of enzymes such as mitogen-activated protein kinase, and improving insulin secretion [21]. The available research results strongly indicate that various carbon sources have different effects on the *G. lucidum* fruiting body growth [22,23,24]. Therefore, this study aims to investigate the composition, functional group characteristics, molecular weight distribution, antioxidant activity in vitro, and growth effect of *L. rhamnosus* LGG of *G. lucidum* polysaccharides under cassava stalk stress. The structure and biological activity of GLPs with different cassava stalk stresses is characterized by gas chromatography-mass spectrometry, near-infrared spectroscopy, and gel chromatography. The results are expected to be of great significance for cassava stalks to be used as a carbon source in the cultivation of *G. lucidum* to realize value-added utilization and to provide basic data support.

## 2. Materials and Methods

### 2.1. The Cultivation of G. lucidum

In this study, *G. lucidum* (Ganoderma lingzhi) was cultivated according to the method proposed by Liu et al. [25], and the relevant parameters were modified appropriately. Cassava stalk (*X*) with *X* values of 30, 40, 50, 60, 70, and 80%, cottonseed hull (83-*X*), wheat bran 10%, corn flour 5%, gypsum powder 1%, and lime 1% were used as cultivation substrates. The water content of the cultivation substrate was adjusted to 55–60%. Then, the mixture was stirred evenly and bagged to prepare bacterial sticks, which were sterilized at 121 °C for 30 min, cooled to room temperature, and inoculated with 1% cultured *G. lucidum* strain

### 2.2. Materials and Reagents

The standard samples of D-Man, L-Rha, D-Glc, D-Gal, L-Ara, D-Fuc, D-Xyl, and D-Fru were purchased from Shanghai Yuanye Biotechnology Co., Ltd. (Shanghai, China). The DPPH free radical scavenging capacity kit (spectrophotometry), total antioxidant capacity (T-AOC) kit (spectrophotometry), and hydroxyl free radical scavenging capacity kit (spectrophotometry) kits were purchased from Beijing Epxilong Biotechnology Co., Ltd. (Beijing, China). *L. rhamnosus* LGG (*Lactobacillus rhamnosus* LGG) was purchased from the China General Microbiological Culture Collection Center Company (CGMCC, Beijing, China).

### 2.3. Extraction of GLPs

GLPs were extracted according to the method described by Liu et al. [5,26], and the related parameters were modified appropriately. Two hundred grams of *G. lucidum* powder and absolute ethanol was 1:15 (*m/v*), which was added into a round-bottomed distillation flask and then reflux-extracted for 3 h at 45 °C under a vacuum of 13.33 Pa, filtered with a 0.25 mm sieve. The oil, pigment, oligosaccharide, and small molecular substances in raw materials were removed, and the filter residue was collected and then dried at 50 °C for 12 h. The *G. lucidum* residue powders were added into a 5 L beaker, and distilled water with a material–water ratio of 1:15 (*m*/*v*) was added. The powders were extracted in a water bath pot at 90 °C for 2 h and filtered with a 0.25 mm sieve. The supernatant was collected and concentrated to 1/3 of the original solution at 55 °C under a vacuum of 13.33 Pa. All concentrated solutions were centrifuged at 5000 rpm for 5 min, and the upper layer of the GLPs solution was collected. The ethanol concentration of the GLPs solution was adjusted to 80% by adding absolute ethanol. The flocculent GLPs precipitate was collected and then freeze-dried at −40 °C for 48 h. Among them, GLPs with cassava stalks accounting for 30, 40, 50, 60, 70, and 80% of the substrate were named GLP1, GLP2, GLP3, GLP4, GLP5, and GLP6, respectively.

### 2.4. Determination of GLPs Content

The polysaccharide content of the *G. lucidum* fruiting body was determined by the phenol‒sulfuric acid method with reference to the method of Liu et al. [5,26]. A 0.1 mg/mL glucose solution was prepared with glucose as the standard. Glucose solutions (0, 0.1, 0.2, 0.3, 0.4, 0.5, 0.6, 0.7, and 0.8 mL) were put into a 15 mL glass test tube with a stopper; distilled water was added to make up to 1 mL to prepare standard glucose solutions of different concentrations, then 1.0 mL 6% phenol solution and 5.0 mL sulfuric acid were added in turn, shaken for 20 s, mixed well, soaked in a water bath at 100 ℃ for 15 min, quickly cooled to room temperature. The absorbance value (*A*) was measured at 490 nm. The standard curve was established according to the absorbance values of different concentrations of glucose solution *C* as follows: *A* = 0.01***C*** + 0.0017 with *R*^2^ = 0.9994.

Ten milligrams of GLPs were added to a 10 mL centrifuge tube, dissolved in 3 mL of distilled water, shaken for 20 s, mixed well, soaked in boiling water for 2 h, cooled to room temperature, filtered through a 0.45 m organic microporous filter membrane, and brought to 100 mL with distilled water in a volumetric flask. The test solution of GLPs was obtained. According to the above steps, the absorbance value was measured at 490 nm, and the polysaccharide content was calculated according to the curve.

### 2.5. Analysis of Monosaccharide Composition of GLPs

The monosaccharide composition in GLPs was determined by ion chromatography with reference to the method of He et al. [27]. The ion chromatograph (ICS5000, ThermoFisher Company, Waltham, MA, USA) was equipped with a chromatographic column Dionex CarbopacTM PA20 (3 × 150 mm) and an electrochemical detector. A total of 5 mg of sample and 2 mL of 3 mol/L trifluoroacetic acid (TFA) were placed into a 15 mL ampoule bottle, and the sample was hydrolyzed at 120 °C for 3 h. One milliliter of hydrolysis solution was added to a 10 mL centrifuge tube and blown dry with nitrogen. Then, 5 mL of water was mixed well into a 10 mL centrifuge tube; 50 μL of hydrolysate solution and 950 μL of deionized water were placed into a 2 mL centrifuge tube with a pipette, the solution was centrifuged at 12,000 rpm for 5 min, and the supernatant was collected. A sample of 5 μL was pumped into the column at a rate of 0.3 mL/min. The column temperature was 30 °C. Mobile phases A, B, and C in the test process were H_2_O, 15 mmol/L NaOH, 15 mmol/L NaOH, and 100 mmol/L CH_3_COONa, respectively.

### 2.6. Determination of the Molecular Weight Distribution of GLPs

The molecular weight and purity of GLPs were determined by high-performance liquid chromatography (HPGPC) with reference to the method of You et al. [28] and Zhang et al. [29]. GLPs and standards of 5000, 11,600, 23,800, 48,600, 80,900, 148,000, 273,000, 409,800, and 667,800 Da were prepared into 5 mg/mL standard solutions and centrifuged at 12,000 rpm for 10 min. The supernatant was filtered with a 0.22 μm microporous filter membrane. The solution was injected into a series gel column (BRT105-104-102, 8 × 300 mm) of a high-performance liquid chromatograph (LC-10A, Shimadzu, Marlborough, MA, USA) to establish the linear regression equation between the retention time (RT, min) and the peak molecular weight (*M*_p_), weight average molecular weight (*M*_w_) and number average molecular weight (*M*_n_), in which the detector was differential. The calibration curves between molecular weight and retention time were as follows: lg*M*_p_ = −0.1767RT + 11.5 with *R*^2^ = 0.9952), lg*M*_w_ = −0.1884RT + 12.047 with *R*^2^ = 0.9933), and lg*M*_n_ = −0.1746*x* + 11.339 with *R*^2^ = 0.9914.

### 2.7. Determination of the Antioxidant Activity of GLPs In Vitro

Determination of total antioxidant capacity (T-AOC): T-AOC was determined by the FRAP method. Then, 75 µL of 125 g/mL GLPs aqueous solution was sucked into a 1.5 mL tube, and 75 µL of distilled water and 850 µL of chromogenic solution (FRAP reagent kit, Suzhou Grace Biotechnology Co., Ltd., Suzhou, China) were added in turn, stirred and mixed evenly, and then reacted at 25 °C for 10 min. Then, the absorbance value (*A*_1_) was measured at 590 nm. If the absorbance value exceeded 1.8, the sample solution was diluted to a specific multiple (*D*) with distilled water. The blank sample (distilled water) was prepared according to the above steps, and the absorbance (*A*_0_) was measured at 590 nm. All GLPs sample tests were zeroed with distilled water and repeated three times. The total antioxidant capacity of GLPs η_1_ (in μmol Trolox/mL units) was derived as follows: η_1_ = 0.36 × ((A_1_ − A_0_)/A_0_ + 0.0262) × *D*.

Determination of DPPH free radical scavenging ability: A 400 µL 500 µg/mL GLPs solution was put into a 1.5 mL tube, and 600 µL of working solution (DPPH reagent kit, Suzhou Grace Biotechnology Co., Ltd.) was added, mixed evenly by vibration, reacted at 25 °C in the dark for 30 min, and centrifuged at 4000 r/min for 5 min, and the absorbance value (*A*_2_) of the supernatant was measured at 517 nm as the measuring group. A 400 µL 500 µg/mL GLPs solution was put into a 1.5 mL tube, 600 µL of 80% methanol was added, and the solution was mixed evenly by vibration. According to the above steps, the supernatant’s absorbance value (*A*_1_) was measured at 517 nm as the control group. Then, 400 µL 80% methanol and 600 µL working solution were placed into the tube and mixed evenly by vibration. According to the above steps, the supernatant’s absorbance value (*A*_1_) was measured at 517 nm as a blank group. All GLPs sample tests were zeroed with distilled water and repeated three times. The DPPH free radical scavenging rate of GLPs was derived as (η_2_, %) = (1 − (*A*_2_ − *A*_1_)/*A*_0_) × 100%.

Determination of hydroxyl radical scavenging ability: 25 µL of reagent #1 was put into a 1.5 mL tube, and 125 µL of reagent #2, 625 µL of 1000 g/mL GLPs solution, and 125 µL of reagent #3 were added in turn, mixed evenly by vibration, and reacted at 37 °C for 20 min. As the measuring group, the absorbance value (A_2_) was measured at 517 nm. A total of 125 µL of reagent #1 was placed into a 1.5 mL tube, and 125 µL of reagent #2, 625 µL of 1000 g/mL GLP solution, and 125 µL of distilled water were mixed evenly by vibration and reacted at 37 °C for 20 min. As the control group, the absorbance value (*A*_1_) was measured at 510 nm. A total of 125 µL of reagent #1 was placed into a 1.5 mL tube, and 125 µL of reagent #2, 625 µL of distilled water, and 125 µL of reagent #3 were mixed evenly by vibration and reacted at 37 °C for 20 min. As a blank group, the absorbance value (*A*_0_) was measured at 510 nm. All GLPs sample tests were zeroed with distilled water and repeated three times. The hydroxyl radical scavenging rate of polysaccharide η_3_ (in %) was derived as follows: η_3_ = 100% × [*A*_0_ − (*A*_2_ − *A*_1_)]/*A*_0_.

### 2.8. Determination of G. lucidum Polysaccharides by Near-Infrared Spectroscopy

*A* 1 mg/mL GLP aqueous solution was placed on the surface of the gold mirror, a drop of sample solution was dropped, and the solution was measured by a near-infrared analyzer (Thermo Nicolet iN10, Thermo Fisher Scientific, Waltham, MA, USA). The whole measurement process was protected by liquid nitrogen and nitrogen. Before each sample measurement, the same collection parameters were used to collect the background spectrum (collection background) of the clean gold mirror surface, with a scanning range of 400–4000 cm^−1^ and a resolution of 4 cm^−1^.

### 2.9. Effect of GLPs on the Growth of Lactobacillus rhamnosus LGG

Under aseptic conditions, 20 µL of *Lactobacillus rhamnosus* LGG was inoculated on MRS broth medium and anaerobically cultured at 37 ± 1 °C for 12 h to activate the strain. A 1% sterilized polysaccharide solution was added to the liquid medium inoculated with 2% (volume fraction) *Lactobacillus rhamnosus* LGGMRS, and deionized water was added as the blank control and cultured at 37 ± 1 °C for 20 h. Starting from 0 h, the bacterial solution OD_600_ was measured every 2 h, and the growth curve of *Lactobacillus rhamnosus* LGG was drawn.

### 2.10. Statistical Analysis

Excel 2013 software was used to sort out the measured data, Origin 8.0 software for mapping, and SPSS 22.0 software for the significance analysis of differences.

## 3. Results and Discussion

### 3.1. Analysis of Total Sugar and Monosaccharide Content in GLPs

The results of the total sugar and monosaccharide composition of GLPs under cassava stalk stress are shown in Table 1. Table 1 shows that the total sugar content of *G. lucidum* was the highest (4.07%) when the proportion of cassava stalk was 30% (GLP1). With the proportion of cassava stalks increasing from 40 to 80%, the total sugar content of GLPs showed a gradually increasing trend. The results of monosaccharide composition analysis showed that the monosaccharides of GLPs obtained under cassava stalk stress were mainly composed of D-Fuc, GalN, L-Rha, L-Ara, GlcN, D-Gal, D-Glc, D-Xyl, and D-Man. The monosaccharide composition of GLPs differed from that described by Huang et al. [30] and Liu et al. [31] due to the different carbon sources for cultivating *G. lucidum*. With the increased proportion of cassava stalk, D-Gal showed an overall decreasing trend, while D-Xyl showed an overall increasing trend. GlcN had the highest content in GLP6, D-Glc in GLP3, and D-Man in GLP2. The above results indicated that the total sugar and monosaccharide composition of GLPs changed under cassava stalk stress, which regulated the metabolic pathway of GLPs.

### 3.2. NIR Analysis of Different GLPs

**Figure 1:**
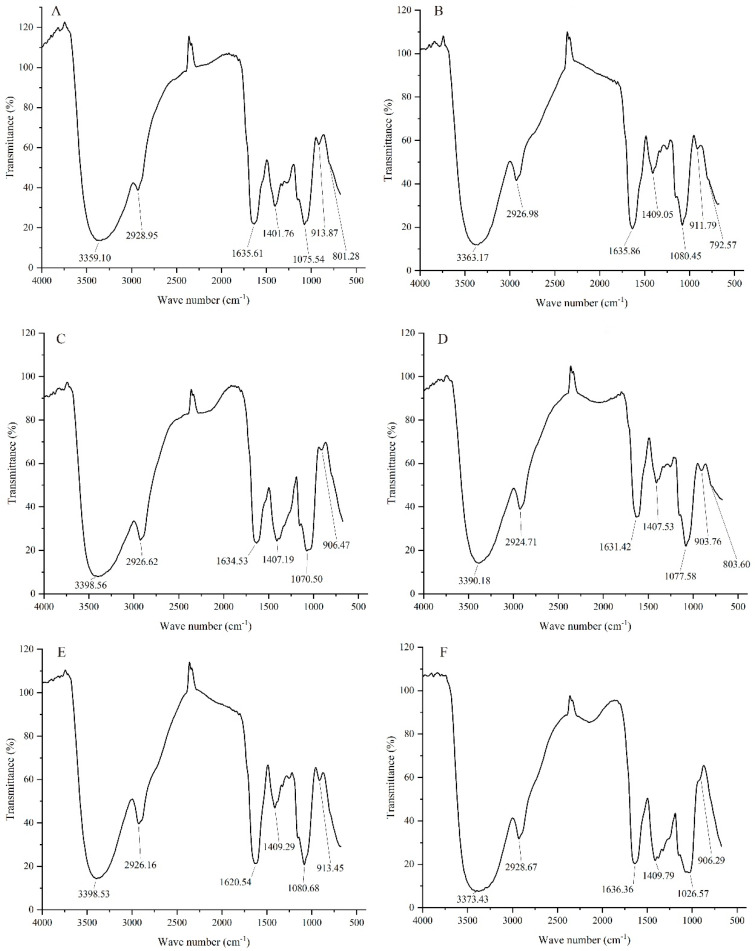
NIR spectra of GLPs under cassava stalk stress: (**A**) GLP1; (**B**) GLP2; (**C**) GLP3; (**D**) GLP4; (**E**) GL5; (**F**) GLP6.

The NIR spectra of GLPs under cassava stalk stress are shown in Figure 1. Figure 1 shows that the characteristic absorption peaks of GLPs range from 3359 to 3398 cm^−1^, 2924 to 2929 cm^−1^, 1620 to 1637 cm^−1^, 1400 to 1410 cm^−1^, 1070 to 1081 cm^−1^, and 903 to 914 cm^−1^. All six GLPs had broad absorption peaks in the range of 3359–3398 cm^−1^, corresponding to O-H stretching vibration [32,33], C-H stretching vibration with a weak absorption peak of alkyl at approximately 2924–2929 cm^−1^ [32], and C=O stretching vibration at 1620–1637 cm^−1^ [34]. That of 1400–1410 cm^−1^ was caused by the variable angle vibration of -CH. The peak near 1048–1084 cm^−1^ was the common resonant absorption peak of the absorption peak of pyranose lactone and hydroxyl groups, which was due to the asymmetric stretching vibration of the C-O-C ether bond on the sugar ring, which constituted the characteristic absorption peak of sugar and the typical infrared spectrum signal of dextran [35]. In addition, 903–914 cm^−1^ featured a typical absorption peak of pyran glucan and β-glycosidic linkage [36], while 876–905 cm^−1^ was a typical absorption peak of β-D-Glc infrared spectrum [34], and 866–914 cm^−1^ was a typical absorption peak of β-D-Gal infrared spectrum [34]. These results strongly indicated that the terminal chains of GLPs were β-D-Glc and β-D-Gal configurations. GLP1, GLP2, GLP3, and GLP5 were β-D-Gal configurations, while GLP4 and GLP6 were β-D-Glc configurations. Combined with the results in Table 1, this implied that the D-Glc content was the highest under the low cassava stalk content, while the D-Glu content increased. The proportion of cassava stalk affected the terminal monosaccharide configuration of GLPs.

### 3.3. Analysis of Molecular Weight Distribution of Different GLPs

**Figure 2:**
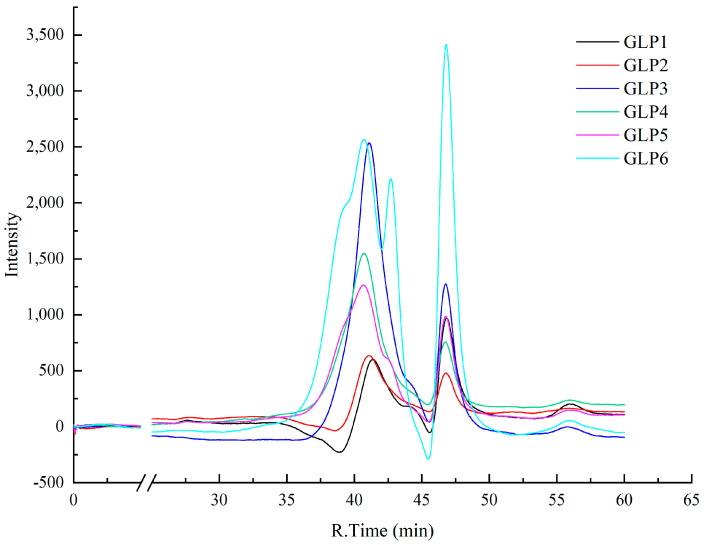
The HPLC gel permeation chromatogram of GLPs under cassava stalk stress, with the mobile phase peak at 46.3 min.

The results of the molecular weight distribution of GLPs under cassava stalk stress are depicted in Figure 2. Figure 2 shows that the peak number, retention time, and strength of GLPs in the gel chromatography column exhibited differences under cassava stalk stress. The molecular weight and other parameters of GLPs with different cassava stalk stresses were calculated via peak molecular weight (*M*_p_), weight average molecular weight (*M*_w_), and number average molecular weight (*M*_n_), as shown in Table 2. According to Table 2, the molecular weight distributions of GLPs with different cassava stalk addition amounts significantly varied. The parameters *M*_w_, *M*_n_, and *M*_p_ were positively correlated, i.e., higher *M*_w_ values corresponded to larger ones *M*_n_ and *M*_p_. The *M*_w_ values of GLPs cultivated by cassava stalk ranged from 6400 to 36,000 g/mol, and the maximum molecular weight of GLPs increased gradually with cassava stalk addition. At cassava stalk proportions exceeding 50 and 70%, the *M*_w_ values exceeded 20,000 and 30,000 g/mol, respectively. The increased proportion of the peak area of high molecular weight polysaccharides indicated that cassava stems could synthesize more high-molecular weight polysaccharides by promoting the growth of *G. lucidum*.

### 3.4. Antioxidant Activity In Vitro Analysis of Different GLPs

**Figure 3:**
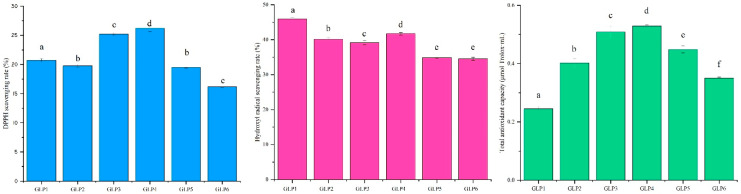
Analysis of antioxidant activity in vivo of GLPs under cassava stalk stress. Different letters a, b, c, d, e and f represents significant differences.

The results of the in vitro antioxidant activity of GLPs under cassava stalk stress are shown in Figure 3. The increased proportion of cassava stalk, DPPH scavenging capacity, and total antioxidant capacity first increased and then dropped, while hydroxyl radical scavenging capacity gradually decreased, and GLP4 had the strongest antioxidant capacity in vitro, followed by GLP3. Dunnett’s T3 significance analysis revealed significant differences in the total antioxidant capacities of GLPs under different cassava stalk stresses (*p* < 0.05). In addition, large differences in DPPH scavenging capacity and hydroxyl radical scavenging capacity were observed between GLP3 and GLP4, as well as between GLP1, GLP2, GLP5, and GLP6.

### 3.5. Effects of GLPs on the Growth of L. rhamnosus LGG

**Figure 4:**
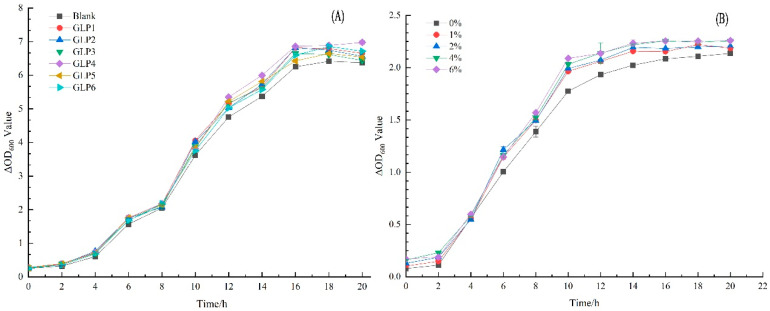
Effect of GLPs under cassava stalk stress on the growth of *L. rhamnosus* LGG. ΔOD represents the absorbance of the *L. rhamnosus* LGG solution.

The effects of GLPs on the growth of *L. rhamnosus* LGG under cassava stalk stress are shown in Figure 4A. Compared with the blank, GLP promoted the growth of *L. rhamnosus* LGG, with 0–4 h as the lag phase, 4–16 h as the logarithmic growth phase, 16–18 h as the stable phase, and 18 h later as the decline phase. The absorbance values of GLP2, GLP3, and GLP4 were larger in the stable phase, which was more conducive to stimulating the growth of *L. rhamnosus* LGG.

To further study the effect of different concentrations of GLPs on the growth curve of *L. rhamnosus* LGG, GLP3 was used as the experimental object, and the growth curve of *L. rhamnosus* LGG with 0–6% GLPs was obtained, as shown in Figure 4B. According to Figure 4B, compared with the blank, increasing the concentration of GLPs was beneficial in stimulating the growth of *L. rhamnosus* LGG. The greater the concentration, the better the stimulation effect. The lag period was 0–2 h, 2–14 h was the logarithmic growth period, and 14–18 h was the stable period. After 18 h, it was the decline period, which promoted the rapid entry of *L. rhamnosus* LGG into the logarithmic growth period to some extent. After 20 h of culture, the growth curves of *L. rhamnosus* LGG stimulated by different concentrations of GLPs partially overlapped, which might be because different samples of GLPs contained components that stimulate different growth stages of *L. rhamnosus* LGG. Overall, when the amount of cassava stalk ranged from 40 to 60%, and the concentration of GLPs was 6%, the growth effect of *L. rhamnosus LGG* was the best.

Noteworthy is that *L. rhamnosus* LGG is the most researched and applied probiotic strain, balancing and improving gastrointestinal function and enhancing human immunity [14]. Therefore, it has been widely applied to developing functional products such as yogurt, milk, juice drinks, etc. [37]. Given the above findings, *G. lucidum* polysaccharide is a lucrative food additive to enhance the functionality of products containing rhamnosus.

## 4. Conclusions

In this study, the structure and biological activity of GLPs with different cassava stalk stresses were characterized by gas chromatography-mass spectrometry, near-infrared spectroscopy, and gel chromatography. The polysaccharide content of *G. lucidum* cultivated with cassava stalk of 1.5–4.0% was determined as the main carbon source. Monosaccharides were mainly composed of D-Fuc, GalN, L-Rha, L-Ara, GlcN, D-Gal, D-Glc, D-Xyl, and D-Man, of which the contents of D-Gal and D-Glc were the largest. β-D-Glc and β-D-Gal were the two main configurations of GLPs under cassava stalk stress. The configuration, molecular weight, and antioxidant activity in vitro of the terminal monosaccharide of GLPs were affected by the proportion of cassava stalk. Higher proportions of cassava stalk corresponded to larger maximum molecular weights of GLPs and higher values of the total antioxidant capacity. Increased concentrations of GLPs promoted the growth of *L. rhamnosus* LGG. However, the mechanism of action on the metabolites of *L. rhamnosus* LGG requires clarification, which is envisaged in a follow-up study.

## Figures and Tables

**Table 1:** Total sugar content and monosaccharide composition of GLPs under cassava stalk stress.

		GLP1	GLP2	GLP3	GLP4	GLP5	GLP6
Total sugar content (%)		4.07 ± 0.01	1.53 ± 0.00	1.83 ± 0.00	2.23 ± 0.02	3.00 ± 0.01	3.34 ± 0.01
D-Fuc	/(mol%)	0.03	0.034	0.024	0.029	0.022	0.012
	μg/mg	9.5	11.6	8.24	8.69	6.08	3.5
GalN	/(mol%)	0	0	0	0	0	0.002
	μg/mg	0	0	0	0	0	0.73
L-Rha	/(mol%)	0	0	0	0	0	0.011
	μg/mg	0	0	0	0	0	3.09
L-Ara	/(mol%)	0	0	0	0	0.029	0.052
	μg/mg	0	0	0	0	7.5	13.5
GlcN	/(mol%)	0.011	0.01	0.008	0.008	0.01	0.013
	μg/mg	4.43	4.63	3.86	3.26	3.7	4.69
D-Gal	/(mol%)	0.36	0.322	0.254	0.26	0.205	0.11
	μg/mg	125.8	120.07	97.64	85.33	63.7	34.5
D-Glc	/(mol%)	0.528	0.552	0.66	0.642	0.643	0.707
	μg/mg	184.57	205.95	253.46	211.06	199.44	221.25
D-Xyl	/(mol%)	0.007	0.007	0.008	0.01	0.035	0.054
	μg/mg	2.1	2.04	2.46	2.84	8.95	14.01
D-Man	/(mol%)	0.065	0.074	0.046	0.05	0.056	0.04
	μg/mg	22.8	27.74	17.51	16.45	17.33	12.41

**Table 2:** Molecular weight parameters of GLPs under cassava stalk stress.

Sample	RT (min)	lg*M*_p_	lg*M*_w_	lg*M*_n_	*M*_p_ (g/mol)	*M*_w_ (g/mol)	*M*_n_ (g/mol)	Peak Area Ratio (%)
GLP1	41.426	4.2	4.2	4.1	15,137	17,472	12,765	84.534
	43.631	3.8	3.8	3.7	6172	6713	5261	15.466
GLP2	41.132	4.2	4.3	4.2	17,060	19,849	14,367	100
GLP3	41.107	4.2	4.3	4.2	17,234	20,065	14,512	93.779
	43.729	3.8	3.8	3.7	5930	6434	5057	6.221
GLP4	40.728	4.3	4.4	4.2	20,108	23,651	16,900	100
GLP5	39.796	4.5	4.5	4.4	29,380	35,435	24,582	32.965
	40.689	4.3	4.4	4.2	20,429	24,054	17,167	50.530
	42.179	4.0	4.1	4.0	11,142	12,603	9431	12.974
	43.629	3.8	3.8	3.7	6177	6719	5265	3.530
GLP6	39.746	4.5	4.6	4.4	29,983	36,212	25,081	29.992
	40.708	4.3	4.4	4.2	20,272	23,857	17,037	41.719
	42.701	4.0	4.0	3.9	9010	10,049	7645	28.289

## Data Availability

The data that support the findings of this study are available from the corresponding author upon reasonable request.
